# Severe Darier's Disease by Mitochondrial DNA Insertion Causing Nonsense Mutations: In Silico Prediction of a Pathophysiological Mechanism to a Novel Mutation

**DOI:** 10.1111/exd.70070

**Published:** 2025-03-10

**Authors:** Haruna Shintani, Yasuaki Ikuno, Hiraku Kokubu, Shino Fujimoto, Akihiko Yamaguchi, Toshifumi Takahashi, Akiko Arakawa, Yukie Kande, Hayato Naka‐Kaneda, Noriki Fujimoto

**Affiliations:** ^1^ Department of Dermatology Shiga University of Medical Science Otsu Shiga Japan; ^2^ Department of Anatomy Shiga University of Medical Science Otsu Shiga Japan; ^3^ Department of Dermatology Ludwig‐Maximilian‐University Munich Germany

**Keywords:** AlphaFold, ATP2A2, Darier's disease, G‐quadruplexes, in silico analysis, mitochondrial DNA insertion, non‐B DNA structure

## Abstract

Darier's disease (DD) is an autosomal dominant genetic disorder caused by mutations in *ATP2A2*. Several cases with nonsense *ATP2A2* mutations presented mild‐to‐moderate phenotypes despite the presumed larger deletion sizes of the ATP2A2 protein. Here, we report a case of severe DD caused by a nonsense mutation with a mitochondrial DNA (mtDNA) insertion despite the smaller presumed deletion size of the ATP2A2 protein. In silico analyses of genomic lesions forming non‐B DNA structures and sequence homology indicated the contingency of this DNA insertion. Analysis of the three‐dimensional structure of the protein predicted no structural disturbance by this insertion. However, the QGRS Mapper algorithm predicted ectopic G‐quadruplex formation in the inserted genome, which may possibly reduce *ATP2A2* transcription. Consistent with this hypothetical mechanism and possible nonsense‐mediated mRNA decay, we identified downregulation of the mtDNA‐inserted *ATP2A2*, which may partially contribute to the severe phenotype in this case. The mtDNA insertions into the human genome are reported to rarely occur, especially in cancers, and only a handful of mtDNA insertions causing genetic diseases are described. This study is the first report to identify mtDNA insertion as a cause of genetic disease in dermatology and demonstrates its pathophysiological mechanism through in silico analyses.

## 1 Background

Darier's disease (DD) is an autosomal dominant disorder characterised by the loss of cell‐to‐cell adhesion and abnormal keratinisation [1]. DD usually presents with hyperkeratotic or crusted papules in a seborrheic distribution and skin folds [2]. The causative gene for DD has been identified as *ATP2A2*, encoding sarco‐endoplasmic reticulum (ER) calcium ATPase type 2 isoform (SERCA2) [3]. SERCA2 plays a crucial role in Ca^2+^ homeostasis by transporting Ca^2+^ from the cytosol to the ER lumen to maintain high Ca^2+^ concentrations in ER [4]. *ATP2A2* mutations identified in DD abolish Ca^2+^ transport by SERCA2, leading to depleted ER Ca^2+^ stores in patient keratinocytes and the subsequent development of DD [5].

Several cases with nonsense *ATP2A2* mutations have been reported [6, 7]. However, these cases were classified as mild‐to‐moderate DD with hyperkeratotic or crusted papules scattered sparsely over the trunk, flexures, or disease limited to some areas [6] despite the deletion of a large part of SERCA2.

Here, we report a case of severe DD presenting with multiple coalescing erythematous hyperkeratotic and crusted papules and pustules involving most of the trunk due to mitochondrial DNA (mtDNA) insertion causing nonsense mutations. Using in silico analyses, we addressed how the insertion occurred and why the patient in our case presented with a severe phenotype, although the deletion size of SARCA2 in our case was much smaller than those in other cases with nonsense mutations. Based on our in silico analyses, we hypothesised that mtDNA insertion may have occurred accidentally and caused the severe phenotype via transcriptional inhibition of *ATPA2* gene through ectopic G‐quadruplex formation. Finally, we tested our hypothesis by confirming the complete loss of mutant *ATP2A2* expression by quantitative polymerase chain reaction (qPCR). This case is the first dermatological genetic disorder with mtDNA insertion, and our results highlight the utility of in silico analysis to predict the pathophysiological mechanisms of novel mutations in genomic disorders.

### 1.1 Questions Addressed

1. How was mtDNA inserted into *ATP2A2*?
2. Why did the patient in our case present with a severe phenotype, although the deletion of a part of SARCA2 in our case was much smaller than in other reported nonsense mutations causing mild‐to‐moderate phenotypes?
3. How can we address the pathophysiological mechanisms of genetic disorders caused by novel mutations?

## 2 Methods

### 2.1 Genomic Sequencing

Peripheral blood samples were obtained from all participants. DNA was extracted from the peripheral blood mononuclear cells. Sequencing of the coding region of the *ATP2A2* gene was analysed by microarray hybrid capture and next‐generation sequencing at the Kazusa DNA Research Institute [8].

### 2.2 Bioinformatics Analysis

To elucidate the mechanism of the insertion, a BLAST search (http://blast.ncbi.nlm.nih.gov/Blast.cgi?CMD=Web&PAGE_TYPE=BlastHome) of the inserted fragment was performed using the Human Genomic Sequences and Transcripts Database (http://www.ncbi.nlm.nih.gov/Tools). The MegaBLAST option, which was designed to identify highly homologous sequences, was used.

The non‐B DNA structure around the insertion site was assessed by non‐B DB database search tools (https://nonb‐abcc.ncifcrf.gov/apps/site/default) [9]. Three‐dimensional (3D) SERCA2 structures have been modelled by AlphaFold [10] using ColabFold [11]. G‐quadruplexes were predicted around the insertion site and in the inserted sequence by the QGRS Mapper algorithm (https://bioinformatics.ramapo.edu/QGRS/index.php) [12].

### 2.3 RNA Isolation and qPCR Analyses

Total RNA was isolated using the Absolutely Total RNA FFPE Purification Kits (Agilent) with treated skin tissues from entire formalin‐fixed paraffin‐embedded sections. Complementary DNA was synthesised by reverse transcription of total isolated RNA (QuantAccuracy RT‐RamDA cDNA Synthesis Kit, TOYOBO, Osaka, Japan). The qPCR for normal *ATP2A2* and the mutant *ATP2A2* with mtDNA insertion and β‐actin (ACTB) was performed using a Step One Plus Thermal Cycler (Applied Biosystems, Foster City, CA) with the THUNDERBIRD Next SYBR qPCR Mix (TOYOBO). Primers used for amplification of specific genes were designed as described in Table 1.

**TABLE 1:** List of qPCR primers.

Target name	Forward primer	Reverse primer	Amplicon size
ACTB	GAGGCACTCTTCCAGCCTTC	TGAAGGTAGTTTCGTGGATGC	70
ATP2A2	TCCCCGGAACCCAAAGGAAC	GGTAGCAGCGCCGACGTAAC	91
Mutant ATP2A2_1	CAAAGGAACCATTGATCAGC	CCAAACCCACTCCACCTTAC	97
Mutant ATP2A2_2	CAAAGGAACCATTGATCAGC	CCCACTCCACCTTACTACCA	92
Mutant ATP2A2_3	CAAAGGAACCATTGATCAGC	ACTACCAGACAACCTTAGCC	79
Mutant ATP2A2_4	TCCCCGGAACCCAAAGGAAC	TCCACCTTACTACCAGACAACC	98

## 3 Results

### 3.1 Clinical Description

A 28‐year‐old Japanese man noticed multiple erythematous papules on his chest and back in 2018. He was diagnosed with multiple folliculitis and was treated with topical antibiotics, oral tetracycline and ultraviolet phototherapy by a local doctor. The erythema remained unchanged, and he was referred to our hospital in 2024.

**FIGURE 1:**
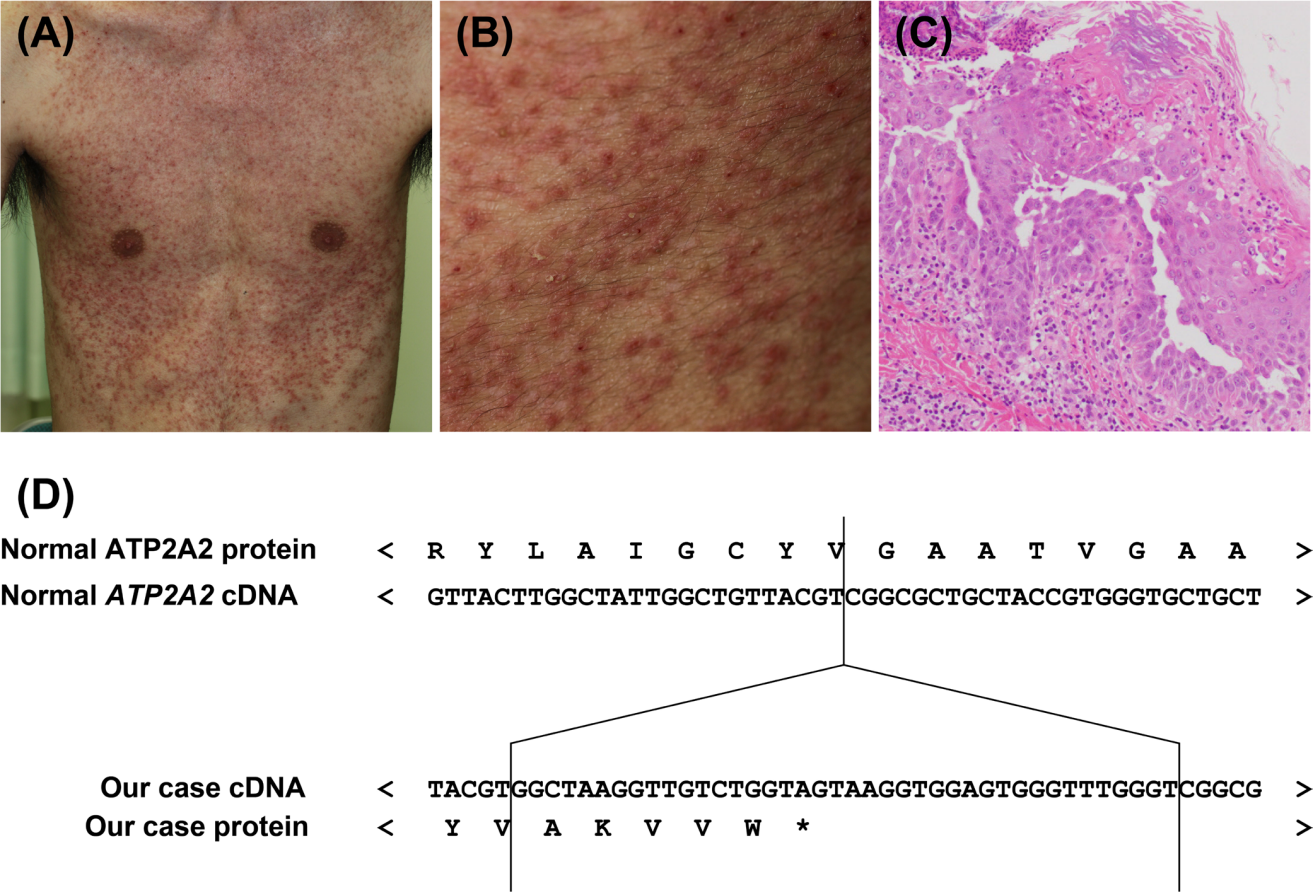
Case presentation. (A, B) Patient presented with multiple coalescing erythematous keratotic papules and pustules on most of his trunk. (C) Histopathological examination of a biopsy specimen from an erythematous pustule showed intracorneal pustules and acantholysis [haematoxylin‐and‐eosin (H&E) staining; magnification, ×100]. (D) Partial sequences of the normal predicted ATP2A2 protein and the corresponding exon, mutant *ATP2A2* gene and the predicted protein in the patient.

He presented with multiple coalescing erythematous hyperkeratotic and crusted papules and pustules on most of his trunk and scattered erythematous papules on his face (Figure 1A,B). He had no relevant medical history. He had no family history of genetic disorders. Laboratory examinations revealed no abnormalities. Histopathological examination of a biopsy specimen from an erythematous pustule showed intracorneal pustules and some Malassezia‐like fungal bodies, acantholysis in the epidermis and perivascular infiltration of inflammatory cells composed of basophils and lymphocytes without perifollicular infiltration of inflammatory cells (Figure 1C). Although the distribution of skin eruptions was not typical, he was suspected of sporadic DD based on the pathological findings.

### 3.2 Sequence Analysis Revealed mtDNA Insertion With Nonsense Mutations in *ATP2A2*

Sequencing of the coding region of the *ATP2A2* gene revealed a heterozygous 40 bp insertion within exon 17, c.2528_2529 ins GGCTAAGGTTGTCTGGTAGTAAGGTGGAGTGGGTTTGGGT (p.Gly844AlafsTer6) (Figure 1D). Using a BLAST search, one sequence yielded a significant alignment, a sequence from the mitochondrial genome (100% homology; ref. NC_012920.1). Therefore, we diagnosed the patient with DD based on mtDNA insertion with nonsense mutations.

### 3.3 In Silico Analysis Indicated Contingency of This DNA Insertion

The mtDNA fragments are usually inserted into the nucleus during double‐strand break (DSB) repair via non‐homologous end‐joining machinery [13, 14]. Non‐B DNA structure formation could lead to DSB [15]. Therefore, we assessed genomic lesions forming a non‐B DNA structure, including a‐phased repeat, direct repeat, G‐quadruplex motif, inverted repeat, mirror repeat and left‐handed Z‐DNA, around the insertion site using non‐B DB database search tools. However, no genomic lesions forming non‐B DNA structures were detected around the insertion site.

No sequence homology was found between the insertion site in the *ATP2A2* gene and the mitochondrial genome, which could explain the integration of the mtDNA fragment at this position. Therefore, it was considered that mtDNA insertion occurred not by any cause but by accidental DSB.

### 3.4 In Silico Analysis Predicted That a Severe Phenotype Was Not Caused by Altered Functional SERCA2 Structure but by G‐Quadruplex Formation in the Inserted Site

Our case presented with multiple coalescing erythematous hyperkeratotic and crusted papules and pustules involving most of the trunk, whereas DD typically presents with scaly papules only in a seborrheic distribution and skin folds [1, 2]. Our case was classified as severe DD based on a previous report [6]; therefore, to confirm whether this severe phenotype was reasonable, three reported cases with nonsense mutations (p. Y122*, p. I521*, p. R528* and p. W551*) were compared, whose symptoms were described alongside our case. Two patients from a single family (p. R528*) and one patient (p. I521*) developed mild DD with hyperkeratotic papules scattered sparsely over the trunk or flexures or disease limited to one or two areas, and two patients in one family (p. Y122*) and one patient (p. W551*) developed moderate DD with more extensive papular lesions or localised verrucous plaques [6, 7]. Our case developed DD in a wider distribution, although the deletion of part of SARCA2 in our case was much smaller than in other cases with nonsense mutations. It was hypothesised that extra peptides disrupted the whole protein structure, leading to loss of function in SERCA2.

**FIGURE 2:**
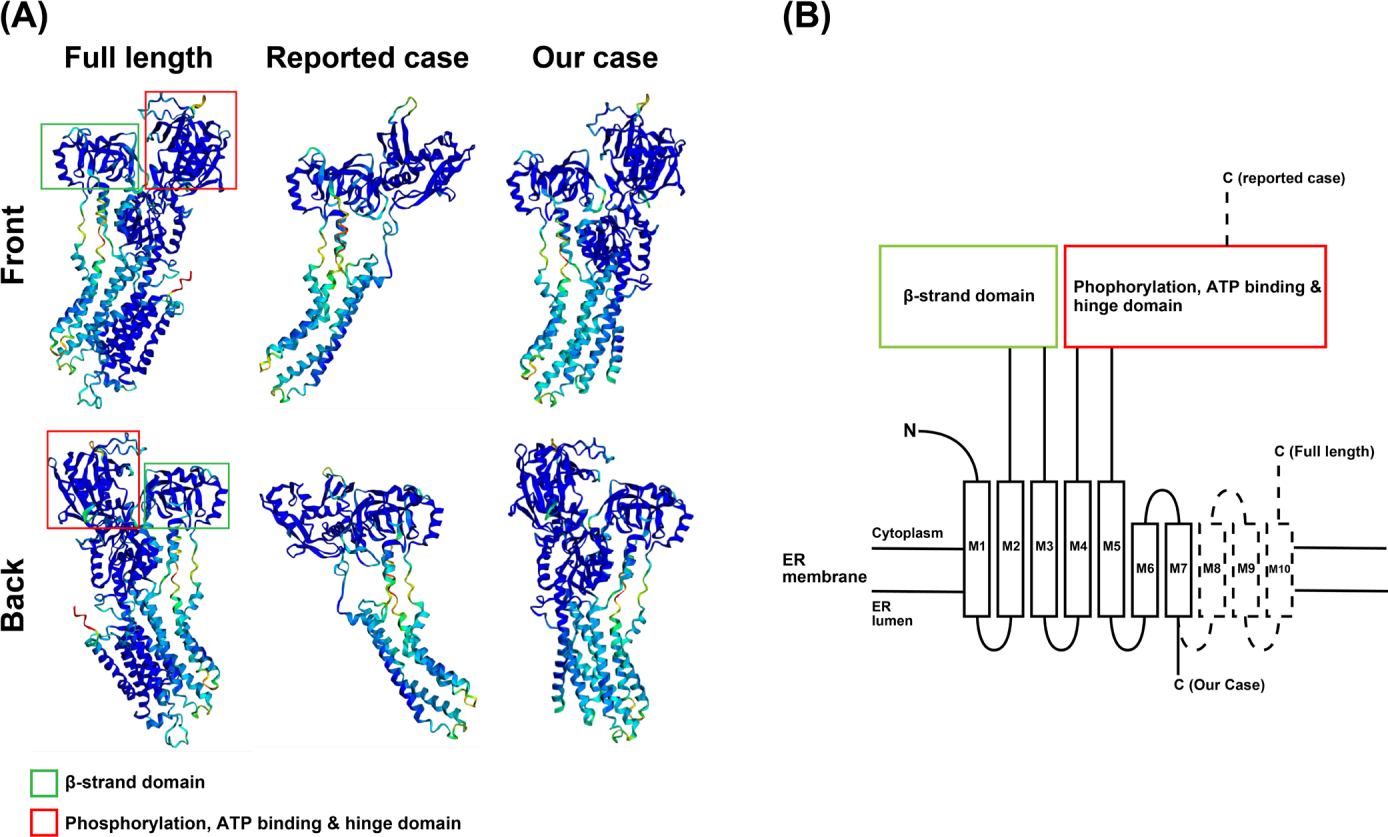
Prediction of the 3D structure of SERCA2 using AlphaFold and qPCR results. (A) Modelled 3D structure of SERCA2 in our case, a previously reported case of a nonsense mutation (p. I521*) and full length. (B) Simple schematic of the SERCA2 domain structure.

To test this hypothesis, we modelled the 3D structure of SERCA2 in our case and a previously reported case of a nonsense mutation (p. I521*) [7] by AlphaFold and compared it with full‐length SERCA2. The structures of the phosphorylation, ATP binding and hinge domains were disrupted in a previously reported case with a mild phenotype. In contrast, the SERCA2 structure in our case was unchanged compared to the full‐length structure (Figure 2A,B). Therefore, we concluded that the severe phenotype observed in our case was not due to a disrupted protein structure.

Next, we focused on the many GG repeats in an inserted sequence, which led to G‐quadruplex structures. The QGRS Mapper algorithm predicted a G‐quadruplex structure (GGTTGTCTGGTAGTAAGGTGGAGTGG) in the inserted sequence. Whether G‐quadruplex structures act as transcriptional activators or repressors depends on the case [16]; however, it was considered that this artificial insertion acted as a transcriptional repressor, and downregulation of *ATP2A2* may have led to a severe phenotype in our case.

**FIGURE 3:**
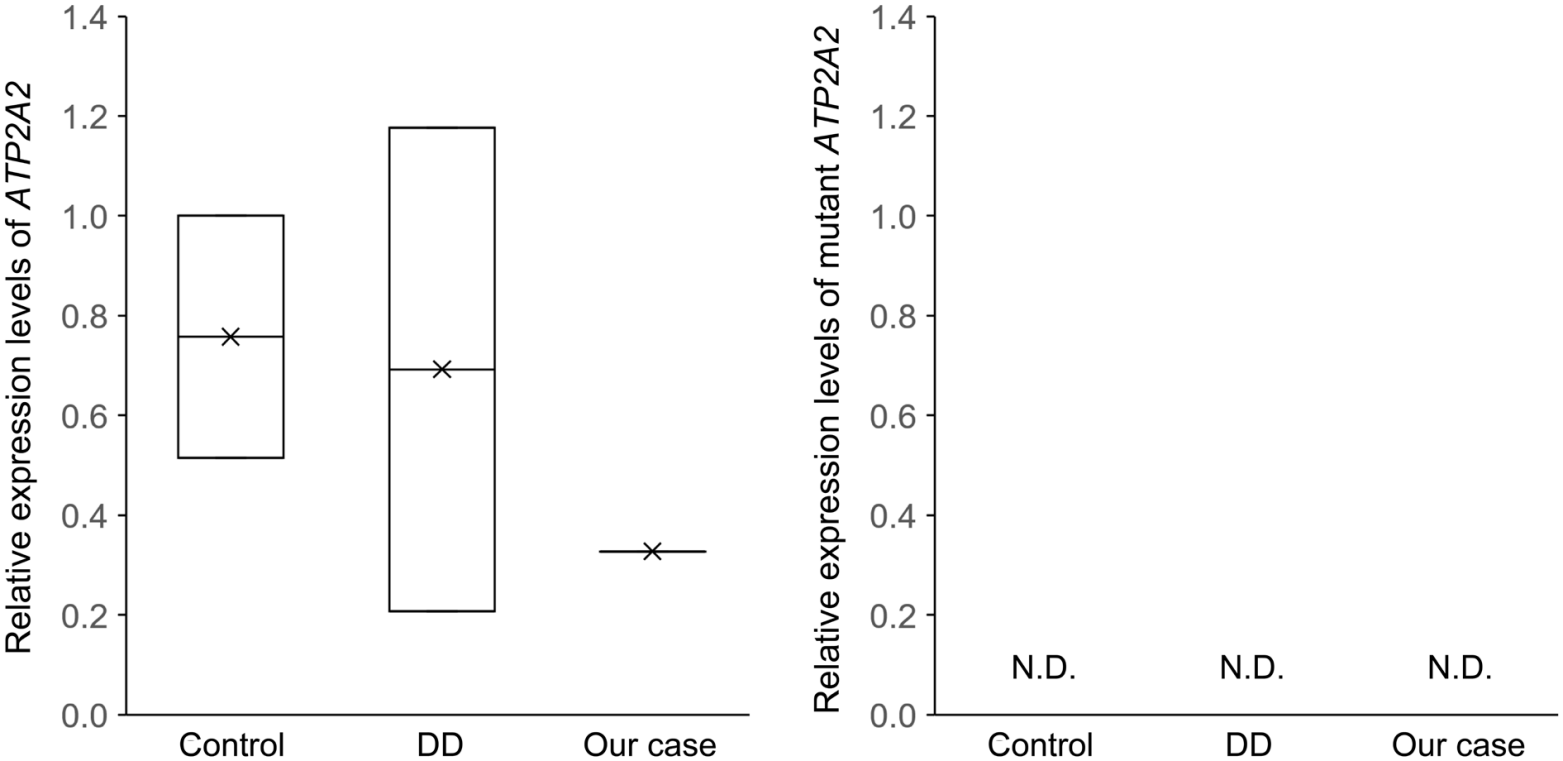
Expression levels of normal *ATP2A2* and the mutant *ATP2A2* with mtDNA insertion in normal skin (Control, *n* = 2), DD with missense mutations (DD, *n* = 2) and in our case (Our case), as assessed by qPCR. The expression of the mtDNA‐inserted mutant *ATP2A2* was completely undetectable (N.D.) in all samples in all four primer sets.

To confirm whether this downregulation of *ATP2A2* occurred, we quantified the expression levels of normal *ATP2A2* and the mutant *ATP2A2* with mtDNA insertion in normal skin, DD with missense mutations, and in our case by qPCR. The expression levels of normal *ATP2A2* in our case were approximately half of those in normal skin and DD with missense mutations. Notably, the expression of the mtDNA‐inserted mutant *ATP2A2* was completely undetectable in all samples by using carefully designed four primer sets (Figure 3). These results suggest that only normal *ATP2A2* expression from one allele was observed, whereas the expression of the mutant *ATP2A2* with inserted mtDNA was completely inhibited in our case, supporting the prediction by in silico analyses.

## 4 Conclusions and Perspectives

Previous studies of various *ATP2A2* mutants have revealed that they all affect SERCA2 function by either decreasing protein expression, Ca^2+^‐ATPase activity and Ca^2+^ transport or altering protein kinetic properties [17]. Truncated SERCA2 by nonsense mutations (p. K542*, p. Q790* and p. E917*) had approximately one‐third of the calcium pump activity compared to the wild type and was greatly reduced by the proteasome due to the deletion of the N‐terminal domain [18]. In addition, the phenotype in our case would be exacerbated by the inserted G‐quadruplex structure, leading to decreased mRNA expression. Further in vitro investigations are required to elucidate the impact of the inserted G‐quadruplex structure on *ATP2A2* mRNA expression.

DNA transfer from mitochondrial DNA to the cell nucleus is ongoing in humans, contributing not only to a complex nuclear–mitochondrial segments landscape, but also, in rare cases, to pathogenic mechanisms of diseases [19]. This study presents an extremely rare DD case of mtDNA insertion causing nonsense mutations in *ATP2A2*. The only reported cases of genetic diseases caused by mtDNA insertion are plasma factor VII deficiency, Pallister‐Hall syndrome, type IV mucolipidosis, Usher syndrome type IC and lissencephaly [20, 21, 22, 23, 24]. To our knowledge, this is the first report of a dermatological genomic disease caused by mtDNA insertion.

Among the reported cases of mtDNA insertion, only one study utilised in silico tools to analyse RNA secondary structures and putative splice sites [24]. We predicted that mtDNA insertion occurred accidentally and that a severe phenotype may be due to downregulation of *ATP2A2* by G‐quadruplexes structure in the inserted site using multiple in silico analyses, including a novel protein prediction method, AlphaFold. In addition to this prediction, nonsense‐mediated mRNA decay, in which mRNAs with translation termination codons are degraded, would contribute to diminished expression of *ATP2A2* with mtDNA insertion [25]. However, nonsense‐mediated mRNA decay would be observed in other cases of DD with nonsense mutations, and the pathophysiology and severity of Darier's disease are mainly attributed to haploinsufficiency and environmental factors, respectively; therefore, the contribution of G‐quadruplexes structure to the severity of DD is still ambiguous [2, 26]. To clarify the precise contribution of G‐quadruplexes and nonsense‐mediated mRNA decay to this diminished expression and the severity of DD, further investigations of DD with nonsense mutations are required.

As the first case, we clarified that mtDNA insertion caused genetic diseases in dermatology using in silico prediction of a pathophysiological mechanism. This report highlights the utility of in silico analyses for predicting pathophysiological mechanisms of novel mutations in genomic disorders.

## Author Contributions

Haruna Shintani, Yasuaki Ikuno, Akihiko Yamaguchi, Akiko Arakawa and Hayato Naka‐Kaneda contributed to the design of the report and drafted the manuscript. Yasuaki Ikuno performed all in silico analyses. Yasuaki Ikuno, Shino Fujimoto, Yukie Kande and Hayato Naka‐Kaneda contributed to RNA isolation and qPCR analyses. All the authors contributed to the design of this study. Toshifumi Takahashi contributed to the revision of the pathology. Yasuaki Ikuno, Hiraku Kokubu, Akihiko Yamaguchi, Toshifumi Takahashi, Akiko Arakawa, Hayato Naka‐Kaneda and Noriki Fujimoto critically revised the manuscript.

## Conflicts of Interest

The authors declare no conflicts of interest.

## Data Availability

The authors have nothing to report.
